# Calcium ions and osteoclastogenesis initiate the induction of bone formation by coral-derived macroporous constructs

**DOI:** 10.1111/jcmm.12125

**Published:** 2013-09-23

**Authors:** Roland M Klar, Raquel Duarte, Therese Dix-Peek, Caroline Dickens, Carlo Ferretti, Ugo Ripamonti

**Affiliations:** a Bone Research Laboratory School of Physiology Faculty of Health Sciences, University of the WitwatersrandJohannesburg, South Africa; b Department of Internal Medicine, University of the WitwatersrandJohannesburg, South Africa

**Keywords:** calcium channel blocker verapamil hydrochloride, osteoclast inhibitor biphosphonate zoledronate, Ca^++^, functionalized nano-patterned topographies, qRT-PCR

## Abstract

Coral-derived calcium carbonate/hydroxyapatite macroporous constructs of the genus Goniopora with limited hydrothermal conversion to hydroxyapatite (7% HA/CC) initiate the induction of bone formation. Which are the molecular signals that initiate pattern formation and the induction of bone formation? To evaluate the role of released calcium ions and osteoclastogenesis, 7% HA/CC was pre-loaded with either 500 μg of the calcium channel blocker, verapamil hydrochloride, or 240 μg of the osteoclast inhibitor, biphosphonate zoledronate, and implanted in the *rectus abdominis* muscle of six adult Chacma baboons *Papio ursinus*. Generated tissues on days 15, 60 and 90 were analysed by histomorphometry and qRT-PCR. On day 15, up-regulation of *type IV collagen* characterized all the implanted constructs correlating with vascular invasion. Zoledronate-treated specimens showed an important delay in tissue patterning and morphogenesis with limited bone formation. Osteoclastic inhibition yielded minimal, if any, bone formation by induction. 7% HA/CC pre-loaded with the Ca^++^ channel blocker verapamil hydrochloride strongly inhibited the induction of bone formation. Down-regulation of *bone morphogenetic protein-2 (BMP-2)* together with up-regulation of *Noggin* genes correlated with limited bone formation in 7% HA/CC pre-loaded with either verapamil or zoledronate, indicating that the induction of bone formation by coral-derived macroporous constructs is *via* the BMPs pathway. The spontaneous induction of bone formation is initiated by a local peak of Ca^++^ activating stem cell differentiation and the induction of bone formation.

## Introduction

The future of the continuing evolution of biomaterials 1 is to functionalize the implanted biomaterials surfaces by activating the surface biology to directly induce specific molecular and tissue biology phenomena initiating regenerative responses as inductive biomaterials 2–12. Self-inductive biomaterials *per se*, without the exogenous application of soluble molecular signals, trigger the ripple-like cascade of pattern formation and tissue induction initiating the generation of form and function, or morphogenesis 3–14.

The basic bone tissue engineering paradigm is tissue induction and morphogenesis by combinatorial molecular protocols whereby soluble molecular signals are re-combined with insoluble signals or substrata acting as tridimensional constructs for the initiation of *de novo* tissue induction and morphogenesis 15–21. The paradigm has been modified by the language of geometry 4–22; a number of systematic studies in heterotopic sites of the Chacma baboon *Papio ursinus* have shown that the driving force of the intrinsic osteoinductivity by bioactive biomaterial matrices is the shape and surface characteristics of the implanted scaffold 4–5. The language of shape is the language of geometry; the language of geometry is the language of a sequence of repetitive concavities that biomimetize the remodelling cycle of the primate osteonic bone 5–24. This has resulted in a hydroxyapatite-coated titanium implant endowed with the intrinsic capacity of inducing bone formation as a result of functionalized tissue-inducing geometric bioreactors constructed along the titanium surfaces 25.

The morphogenesis of bone by calcium phosphate-based macroporous bioceramics when implanted in heterotopic sites was first reported when implanting coral-derived fully converted hydroxyapatite constructs in the *rectus abdominis* muscle of adult non-human primates *P. ursinus* 13–14. Systematic studies in *P. ursinus* were then initiated to further understand the induction of bone formation by coral-derived macroporous constructs harvested at different time periods 3–29. Because of the availability of comprehensive acquired data all obtained in *P. ursinus,* further mechanistic studies were performed in the same animal model.

Which are the molecular signals that set into motion cell differentiation, pattern formation and the induction of bone formation by coral-derived macroporous constructs? To mechanistically further our knowledge on the spontaneous and intrinsic induction of bone formation by coral-derived macroporous constructs, a series of treated and untreated coral-derived biomimetic matrices were implanted in the *rectus abdominis* muscle of the Chacma baboon *P. ursinus*. Generated tissues at different time-points were harvested and evaluated morphologically, histomorphometrically and by qRT-PCR.

Osteoclastic-driven functionalized nano-patterned topographies with calcium ions (Ca^++^) release re-programmed somatic stem cells to initiate *de novo* bone formation. The induction of *bone morphogenetic protein-2 (BMP-2)* gene expression, the prominent expression of *type IV collagen* pre-dating the induction of bone formation together with down-regulation of *BMP-2* and up-regulation of *Noggin* with corresponding limited bone formation by treated macroporous constructs with the Ca^++^ channels blocker, verapamil hydrochloride and the osteoclast inhibitor, biphosphonate zoledronate, form the basis of this communication.

## Materials and methods

### Macroporous coral-derived calcium carbonate/hydroxyapatite constructs

Macroporous replicas of coral-derived calcium carbonate exoskeletons of the genus Gonipora were prepared by hydrothermal chemical exchange with phosphate 14–30. Limited conversion to hydroxyapatite resulted in calcium carbonate constructs with 7% hydroxyapatite defined as 7% HA/CC (Biomet, Interpore Cross, Irvine, CA, USA). 7% HA/CC constructs were rods 8 mm in diameter and 20 mm in length 29. The solid components of the hydroxyapatite/calcium carbonate replica average 130 μm in diameter and their interconnections 220 μm; the average porosity is 600 μm and their interconnections average 260 μm in diameter 14–30.

### Pre-loading of coral-derived constructs with the calcium ion channel blocker, verapamil hydrochloride and the osteoclast inhibitor, biphosphonate zoledronate

Topographical osteoclastic modifications of the 7% HA/CC macroporous surfaces have been suggested to be a critical event initiating the spontaneous induction of bone formation 29. Release of calcium ions by osteoclasts during bone resorption regulates cellular differentiation and induces angiogenesis 31–36. Macroporous 7% HA/CC constructs were loaded with either 240 μg of the biphosphonate zoledronate (Zometa®, Novartis, Kempton Park, Johannesburg, South Africa), an osteoclast inhibitor analogue, or 500 μg of verapamil hydrochloride (Isoptin®, Knoll Pharmaceutical, Port Elizabeth, South Africa), an L-type voltage gated calcium channel blocker. Untreated 7% HA/CC constructs were used as control. Under laminar flow, pre-loading of the re-suspended inhibitors was by pipetting the required amount of liquid vehicle onto both proximal and distal regions of the 7% HA/CC constructs to ensure an even distribution of the various added components throughout the macroporous spaces.

### Primate model for tissue induction and morphogenesis

**Figure 1:**
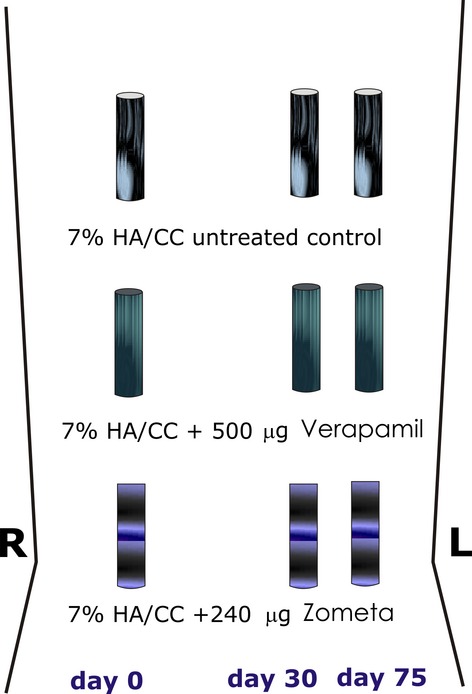
Heterotopic intramuscular *rectus abdominis* surgical model and implantation design in six adult non-human primates Chacma baboon *Papio ursinus*. In each animal, a total of nine coral-derived calcium carbonate macroporous constructs with limited hydrothermal conversion to hydroxyapatite (7% HA/CC) were implanted heterotopically in triplicate. Three 7% HA/CC constructs representing one treatment modality each were implanted at days 0, 30 and 75, thus providing tissue constructs with invading differentiating tissues to be harvested at days 90, 60 and 15 after heterotopic implantation. Implants were aligned longitudinally ∼8 and 2 cm apart.

Six clinically healthy adult *P. ursinus* with a mean weight of 21.2 (±1.48) kg were selected from the primate colony of the University of the Witwatersrand, Johannesburg. Criteria for selection, housing conditions and diets were as described 14. *Papio ursinus* species share similar bone physiology and osteonic bone remodelling with humans 37. Research protocols were approved by the Animal Ethics Screening Committee of the University, and conducted according to the *Guidelines for the Care and Use of Experimental Animals* prepared by the University and in compliance with the National Code for Animal Use in Research, Education and Diagnosis in South Africa 38. Premedication, before induction of general anaesthesia, was by midazolam hydrochloride (Dormicum® 3 mg/kg IM, Roche, Illovo, South Africa). Animals were anaesthetized with ketamine hydrochloride (5–15 mg/kg; Eutaphent Kyron Laboratories, Johannesburg, South Africa) and general anaesthesia maintained by Isofor (1.5–2%; Safe Line Pharmaceutical, Johannesburg, South Africa), after oro-tracheal intubation 28–29. A total of nine 7% HA/CC untreated and treated macroporous constructs were implanted bilaterally in intramuscular pouches surgically created by sharp and blunt dissection within the *rectus abdominis* muscle of each animal (Fig. 1), three on day 0, three on day 30 and three on day 75. Implants were aligned longitudinally ∼2 cm apart 29. Post-operative pain and inflammation were controlled by buprenorphine hydrochloride (Temgesic 0.3 mg/kg IM), and carpofan (Rimadyl 3 mg/kg *subcutis*).

### Tissue harvest, histology and histomorphometry

On day 90, animals were anaesthetized with an injection of ketamine (5–15 mg/kg) and maintained under general anaesthesia with Isofor (1.5–2%). Specimens were harvested together with muscle tissue (baseline control). Following harvest, animals were killed with an intravenous overdose of sodium pentobarbitone (Anaket, Isando, South Africa). Harvested specimens provided regenerating tissues at 15, 60 and 90 days after intramuscular implantation. A 4 mm fragment of each harvested 7% HA/CC construct with invading differentiating tissues within the macroporous spaces was cut with sterile blades, and flash frozen in liquid Nitrogen. Remaining tissues were fixed in 10% phosphate buffered formalin for 48 hrs, rinsed in tap water and stored in 70% ethanol 29–30. Specimens were demineralized in a Sakura TDE™ 30 decalcifying unit (Sakura, Fintek, Torrance, CA, USA) and processed for paraffin wax embedding. Thirty sections were cut sequentially at 4 μm to obtain a full cross-sectional area of each specimen. The 1st and every 10th section was stained with a modified Goldner's Trichrome stain for histological and histomorphometric analyses 28–29.

Images of the histological sections were captured and digitalized with a Provis AX70 research microscope (Olympus Optical Co., Shinjuku, Tokyo, Japan) attached to a video camera (Olympus). Histomorphometric analysis was then performed with the Stream Essentials software v.1.6 (Olympus). The *ratios* of the total area of the devices and the area of newly formed bone generated within the evaluated macroporous spaces were established, and values were expressed, as a mean percentage of bone generated within the evaluated macroporous spaces. Results were analysed with a Student's *t*-test using STATISTICA version 10 (Statsoft Inc., Tulsa, OK, USA).

### qRT-PCR

Under sterile conditions, redundant soft tissue was removed from heterotopic specimens, and the 4 mm fragments of each harvested specimens were then ground to powder in liquid Nitrogen with a mortar and pestle. Total RNA was extracted from 30 mg of pulverized specimen with the Total RNA Mini Kit (Bio-Rad, Hercules, CA, USA). RNA concentration was determined spectrophotometrically at A_260/280_ with a Nanodrop 2000 (Thermo Scientific, Waltham, MA, USA) and RNA quality was assessed with a Pico6000 RNA kit (Agilent Technologies, Santa Clara, CA, USA) on a Bioanalyzer 2100 (Agilent Technologies). RNA integrity numbers were between 8.0 and 9.0. RNA was reverse transcribed with the High Capacity cDNA Reverse Transcription kit (Applied Biosystems, Foster City, CA, USA). qRT-PCR was performed, in duplicate with EvaGreen Master Mix (Bio-Rad) on a CFX-96 thermocycler (Bio-Rad). Each reaction contained 10 ng cDNA; 1× EvaGreen Mix and 10 μM of each primer (Table 1). Primers were designed with Gene fisher v. 2.0 (http://bibiserv.techfak.uni-bielefeld.de/genefisher2). Thermocycling parameters included a denaturation step of 94°C for 2 min.; 40 cycles of 95°C for 10 sec., 60°C for 10 sec. and 72°C for 30 sec.; and a final extension at 72°C for 5 min. Genes assayed included *BMP-2; Type IV Collagen* and *Noggin*. Gene expression was normalized against four reference genes: *ß-Actin; Succinate dehydrogenase complex subunit A (SDHA); Ribosomal Protein L13A (RPL13a) and Ribosomal Protein Large PO (RPLPO*). Use of GeNorm (http://medgen.ugent.be/∼jvdesomp/genorm/) established that these were the most appropriate internal reference genes to use. Amplified PCR products underwent sequencing (Inqaba Biotech, Pretoria, South Africa) to confirm that the correct sequence had been amplified.

**Table 1:** Primer sequence

Gene	Primer Sequence (5′–3′)
*BMP-2*	F: AGTTGCGGCTGCTCAGCATGTT R: ACATGTCTCTTGGAGACACCT
*Collagen type IV*	F: GTTGGTCTACCGGGACTCAA R: GTTCACCTCTGATCCCCTGA
*Noggin*	F: GAGGAAGTTACAGATGTGGCT R: CACTCGGAAATGATGGGGTAC
*SDHA (reference)*	F: TGGGAACAAGAGGGCATCTG R: CCACCACTGCATCAAATTCATG
*RPL13a (reference)*	F: CCTGGAGGAGAAGAGGAAAGAGA R: TGAGGACCTCTGTGTATTTGTCAA
*β-actin (reference)*	F: CTCTTCCAGCCTTCCTTCCT R: AGCACTGTGTTGGCGTACAG
*RPLP0 (reference)*	F: AGCTGATCAAGACTGGAGACA R: TCCAGGAAGCGAGAATGCAGAGTT

Gene expression from the harvested macroporous devices was normalized to muscle tissue in each animal. Calibrated normalized relative quantities (CNRQs), which reflect the log_10_^−ΔΔC*t*^, were determined with qBase analysis software (http://www.biogazelle.com). A Student's *t*-test (STATISTICA version 10, Statsoft Inc.) was used to determine statistical significance at *p* < 0.05.

## Results

### Bone formation by autoinduction in 7% HA/CC untreated macroporous constructs

**Figure 2:**
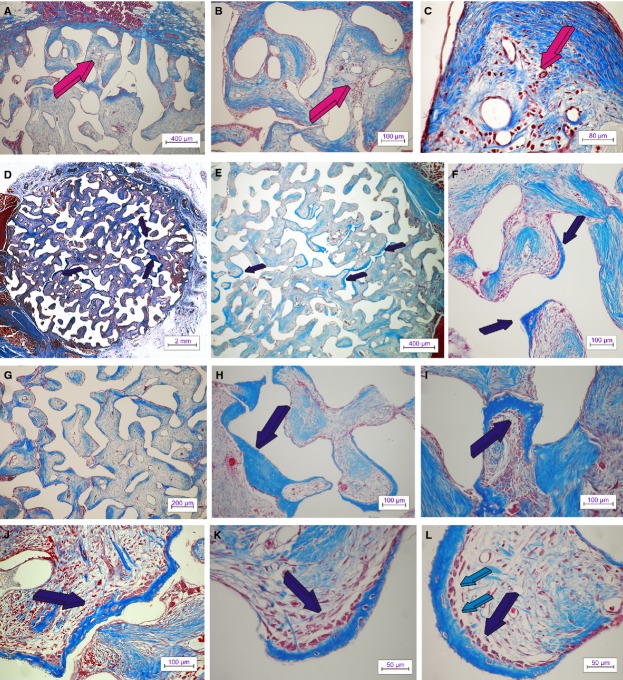
Untreated control 7% HA/CC coral-derived macroporous constructs on days 15 and 60 after heterotopic intramuscular implantation. Macroporous self-inducing geometric cues of untreated coral-derived biomimetic constructs induce angiogenesis with capillary sprouting and invasion, cellular trafficking, pattern formation and the morphogenesis of collagenous condensations attached to the macroporous surfaces facing a highly vascular penetrating mesenchymal tissue (magenta arrows in A–C) as early as 15 days after intramuscular implantation. By day 60 (D–H), there are further remodelling and tissue patterning with the induction of bone formation (dark blue arrows). Bone preferentially forms by induction within concavities of the 7% HA/CC macroporous constructs (dark blue arrows in F, H, K and L). Newly formed bone by induction with embedded osteocytes is tightly connected to the implanted biomatrix (I–L); the newly induced bone matrix is surfaced by plump contiguous secreting osteoblasts facing a highly angiogenic mesenchymal supporting matrix (dark blue arrows in K and L). Note how capillary basement membranes touch osteoblastic cells lining newly formed bone within concavities of the substratum (large arrow in L). (Decalcified sections cut at 4 μm stained with Goldner's trichrome).

Digital iconographic images of 7% HA/CC untreated control are presented in Figure 2, at 15 and 60 days, and Figure 5, at 90 days. At harvest, all implants were firmly attached to the ventral fascia and the surrounding *rectus abdominis* muscle. On day 15, the most peripheral macroporous spaces were invaded by a highly vascular connective tissue matrix (Fig. 2A–C). Vascular invasion and capillary sprouting was pronounced in all treatment modalities. Previous experiments in the non-human primate *P. ursinus* have shown that the specific geometry and surface characteristics of the coral-derived substratum are conducive to rapid vessels ingrowths’ and capillary sprouting within the early mesenchyme penetrating the macroporous spaces 3.

**Figure 3:**
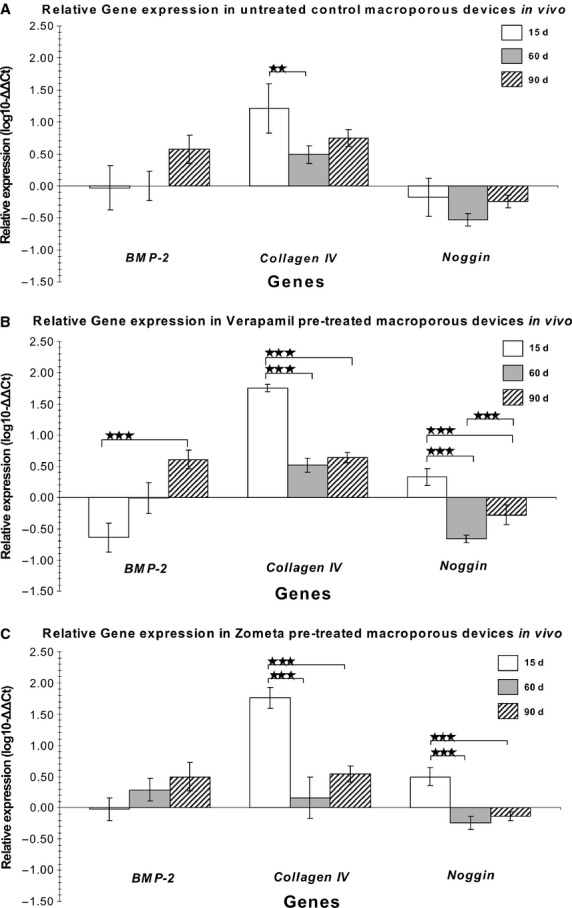
Relative change in gene expression of BMP-2, collagen type IV and Noggin at days 15 (15d), 60 (60d) and 90 (90d) within untreated 7% HA/CC control (A), verapamil (B) and zoledronate-pre-treated 7% HA/CC (C) implanted within the *rectus abdominis* muscle of six adult Chacma baboons *Papio ursinus* (**p < 0.05, ***p < 0.01).

Angiogenesis correlated with *type IV collagen* up-regulation irrespective of the treatment modalities (Fig. 3). 7% HA/CC untreated controls showed pronounced capillary sprouting and vascular invasion within the peripheral macroporous spaces (Fig. 2A–C). Morphogenesis and tissue patterning of collagenous condensations were well advanced on day 15 (Fig. 2B and C) with remodelling and further development on day 60 (Fig. 2D–F). Areas with prominent angiogenesis showed a pronounced capillary sprouting and invasion with hypercellularity (Fig. 2B and C). Paravascular pericytic cells showed enlarged hyper-chromatic nuclei with several cells migrating between the vascular compartment and the paravascular extracellular matrix (Fig. 2C).

**Figure 4:**
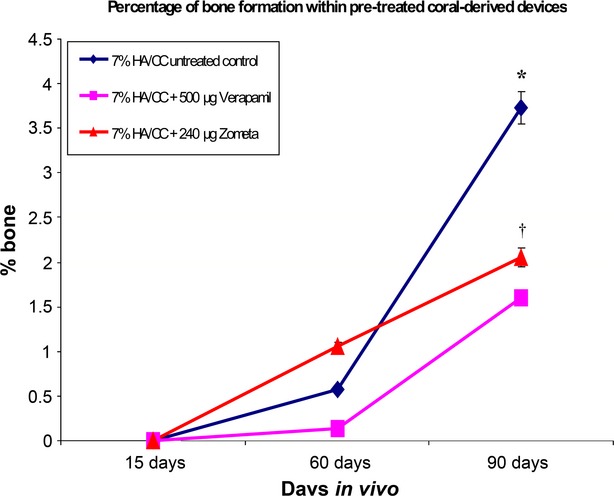
Morphometric analyses of induced bone by 7% HA/CC untreated control *versus* verapamil hydrochloride and zoledronate-treated specimens. Note the pronounced and significant inhibitory effects on the induction of bone formation by both verapamil hydrochloride- and zoledronate-treated specimens *versus* untreated control on day 90 (*p < 0.05). Zoledronate-treated specimens showed the least bone formation by induction on day 90 also when compared with verapamil hydrochloride-treated specimens (†*p* < 0.05).

On day 60, untreated macroporous constructs showed bone formation by autoinduction throughout several macroporous spaces (Fig. 2D–F) correlating with the morphometric analyses (Fig. 4). Histological analyses at high magnification showed that bone was tightly opposed to the macroporous spaces surfaced by contiguous plumped and secreting osteoblasts facing a highly vascularized matrix (Fig. 2H–L). Of note, several areas of newly auto induced bone had formed preferentially in concavities of the substratum (Fig. 2K and L) following a repetitive sequence of events as highlighted by the ‘*geometric induction of bone formation*’ 9,10. Importantly, osteoblastic cells were directly opposed to the basement membranes of the capillaries that had formed within the macroporous spaces as shown in Figure 2L (light blue arrows).

**Figure 5:**
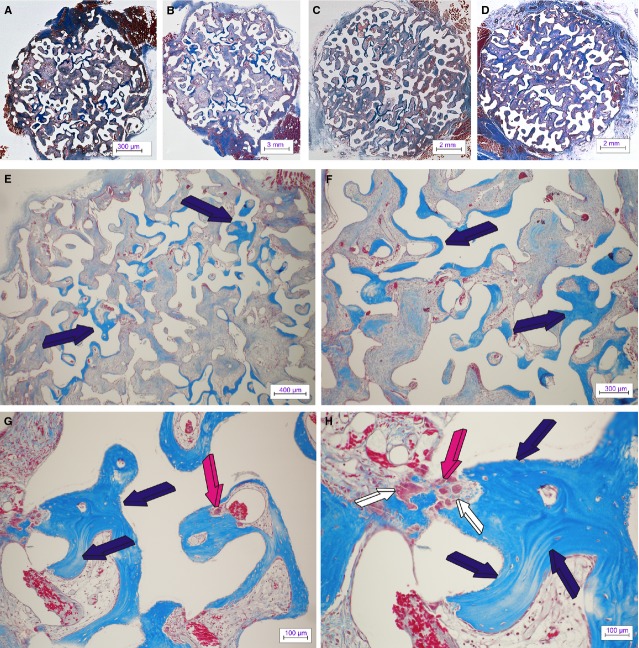
Untreated control 7% HA/CC macroporous constructs on day 90. ‘Spontaneous’ and ‘intrinsic’ bone differentiation and remodelling in untreated control 7% HA/CC biomimetic constructs harvested on day 90 after intramuscular heterotopic implantation. (A–D) Low power views showing tissue patterning, remodelling of collagenous condensations and bone formation by induction within selected macroporous spaces. (E and F) High power views of previous sections (A and B) showing the induction of bone formation (dark blue arrows) supported by a pronounced vascular invasion across the macroporous spaces. (G and H) There is prominent remodelling with osteoclastogenesis (magenta arrows) of the newly formed corticalized lamellar bone (dark blue arrows). Note the intimacy of vascular invasion with newly differentiated large multinucleated osteoclastic cells within the remodelled bone matrix (white arrows). Decalcified sections cut at 4 μm stained with Goldner's trichrome.

By day 90, auto induced bone by untreated macroporous constructs increased significantly (Fig. 4), and remodelled in blocks of osteonic lamellar bone throughout the macroporous spaces (Fig. 5A–F) with osteocytes embedded within the newly formed matrix (Fig. 5G and H). Capillary sprouting was associated with osteoclastic activity remodelling the newly formed bone (Fig. 5H).

### Biphosphonate zoledronate-treated macroporous constructs

**Figure 6:**
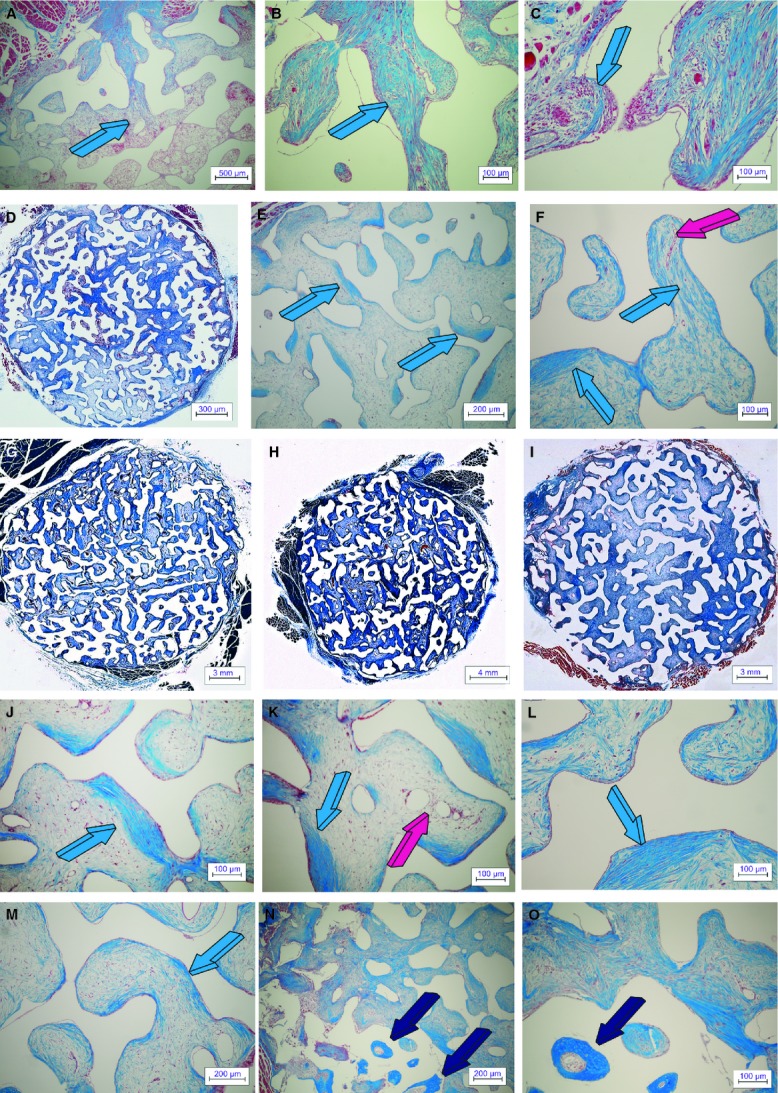
Zoledronate-treated 7% HA/CC macroporous constructs on days 15, 60 and 90. Tissue patterning and morphogenesis of fibrovascular mesenchymal tissue penetrating the macroporous spaces of biphosphonate zoledronate-treated macroporous 7% HA/CC constructs. The angiogenic fibroblastic front advances within the interconnected macroporous spaces invading the loose vascular matrix suspended by the remaining fibrin/fibronectin scaffold that has formed within the macroporous spaces (light blue arrow in A). Tissue patterning and morphogenesis of collagenous condensations not yet tightly attached to the biomimetic matrix (light blue arrows in B and C). (D–F) Remodelling, tissue patterning and morphogenesis of collagenous condensations against the implanted biomatrix on day 60 is still profoundly delayed when compared with tissue invasion and morphogenesis as evaluated in untreated control specimens even when harvested on day 15 (Fig. 2A–C). (G–I) Low power views of tissue incorporation and mesenchymal tissue invasion in zoledronate-treated macroporous 7% HA/CC constructs harvested on day 90 showing delayed mesenchymal tissue patterning facing a very loose fibrovascular tissue. (J–M) Delayed tissue patterning and morphogenesis of collagenous condensations against the macroporous constructs (light blue arrows) with still prominent vascular invasion (magenta arrow). (N and O) On day 90, and at the periphery of the macroporous constructs, possibly related to the lack of diffusion of the pipetted biphosphonate zoledronate, there is occasionally the induction of bone formation (dark blue arrows). Decalcified sections cut at 6 μm stained with Goldner's trichrome.

Digital iconographic images of 7% HA/CC biphosphonate zoledronate-treated macroporous constructs are presented in Figure 6, at 15, 60 and 90 days. On day 15, the most peripheral macroporous spaces were invaded by a rather loose and less organized fibro-vascular tissue when compared with untreated control specimens (Fig. 6A) without tissue patterning as in control specimens, which already showed the morphogenesis of mesenchymal collagenous condensations on day 15 (Fig. 2A–C). Collagenous fibres were as yet to be patterned and assembled interacting with a poorly organized fibrovascular tissue with capillary sprouting (Fig. 6B and C).

Collagenous condensations against the macroporous surfaces were seen to be still organizing on day 60 facing a rather loose yet vascular connective tissue matrix (Fig. 6D–F). In general, tissue patterning and morphogenesis were greatly delayed when compared with untreated control specimens. On day 90, collagenous condensations were more organized, but not patterned as in 7% HA-CC untreated control, still facing a rather loose connective tissue matrix (Fig. 6J–M). On day 90 at the periphery of the specimens, there was occasionally the induction of bone formation (Fig. 6N and O).

### Calcium ion channel blocker verapamil pre-treated macroporous constructs

**Figure 7:**
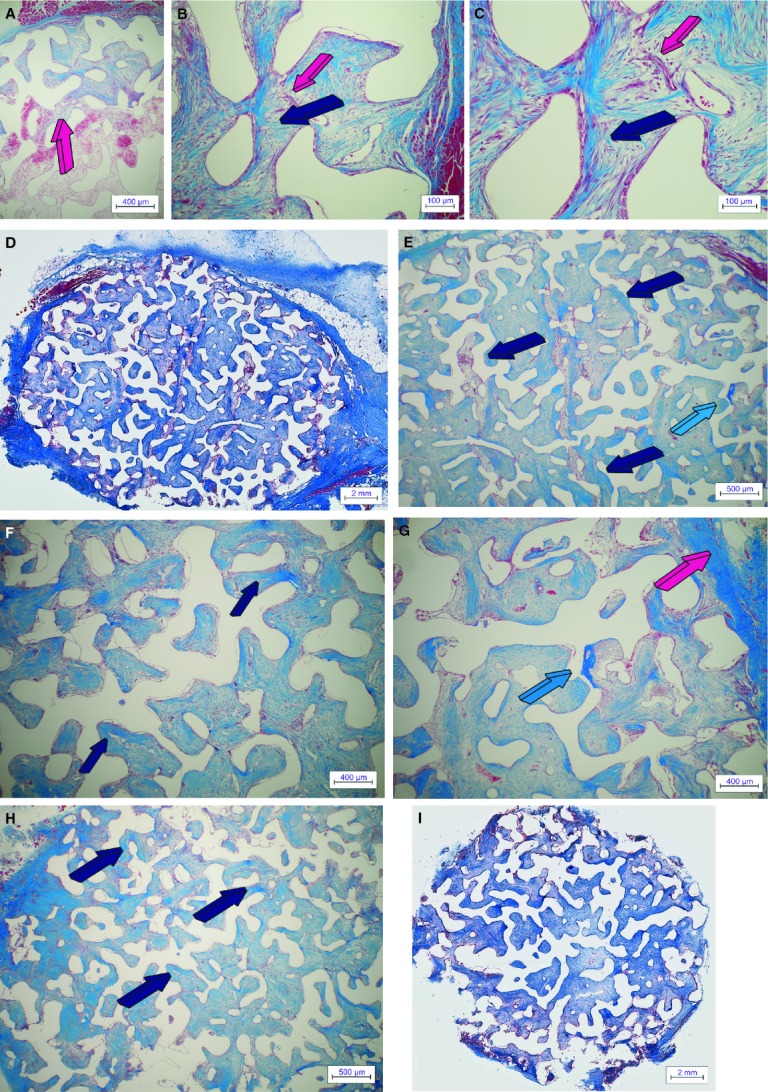
Ca^++^ channel blocker verapamil hydrochloride-treated 7% HA/CC macroporous constructs on days 15, 60 and 90. Tissue induction, morphogenesis and tissue patterning of mesenchymal fibrovascular tissue invasion in 7% HA/CC macroporous spaces pre-loaded with the Ca^++^ channel blocker verapamil hydrochloride implanted in the *rectus abdominis* muscle of *Papio ursinus* and harvested on days 15 (A–C), 60 (D–G) and 90 (H and I). (A) Tissue patterning of fibrovascular tissue invasion on day 15 with fibrovascular bundles invading the loose fibrin-fibronectin vascular matrix (magenta arrow) within the more internal macroporous spaces. (B and C) Early tissue morphogenesis of collagenous condensations (dark blue arrows) supported by a rich vascular invasion (magenta arrows). (D and E) Low power views of a verapamil hydrochloride-treated constructs implanted in the *rectus abdominis* of *P. ursinus* and harvested on day 60. Morphogenesis and tissue patterning of collagenous condensations (dark blue arrows) with the induction of an island of bone at the very periphery of the implanted macroporous construct (light blue arrow in E). (F) Remodelling of the fibrovascular tissue invasion and mesenchymal collagenous condensations (dark blue arrows) and (G) detail of newly formed bone (light blue arrow) at the periphery of the implanted macroporous construct surrounded by the *rectus abdominis* fascia (magenta arrow). (H and I) Delayed tissue patterning and collagenous condensations in Verapamil hydrochloride-treated specimens harvested on day 90. Limited organization and tissue patterning of collagenous condensations with, however, prominent vascularization across the macroporous spaces. Decalcified sections cut at 4 μm stained with Goldner's trichrome.

Digital iconographic images of 7% HA/CC Ca^++^ channel blocker verapamil pre-treated constructs are presented in Figure 7, at 15, 60 and 90 days. On day 15, there was fibrovascular tissue invasion within the most peripheral macroporous spaces with morphogenesis of cellular condensations along the macroporous surfaces of the implanted 7% HA/CC constructs (Fig. 7A–C). Vascular invasion and capillary sprouting were well pronounced (Fig. 7B and C) correlating with the up- regulation of the *type IV collagen* gene (Fig. 3B).

On day 60, there were morphogenesis and remodelling of collagenous condensations against the macroporous spaces (Fig. 7D–F). Minimal bone formation, when found, was only present at the very periphery of the implanted matrix, bordering with the fibro-muscular tissue surrounding the harvested specimens (Fig. 7E and G). Tissue patterning and morphogenesis of mesenchymal collagenous condensations were delayed when compared with untreated control. A thorough morphological analysis of treated and untreated macroporous samples indicated that the greater delays in tissue patterning and morphogenesis of collagenous condensations were seen in macroporous constructs pre-treated with 240 μg of the biphosphonate zoledronate (Fig. 6).

On day 90, the macroporous spaces of Ca^++^ channel blocker verapamil pre-treated constructs were invaded by fibrovascular tissue with scattered condensations throughout the implanted matrix (Fig. 7H and I). Tissue patterning and morphogenesis of collagenous condensations were greatly delayed when compared with untreated control specimens (Figs 2 and 5).

### qRT-PCR

The intensity of the expression together with the relative changes in gene expression of *BMP-2*,*type IV collagen*, and *Noggin* are presented in Figure 3. *Type IV collagen* was up-regulated on day 15 (*p* < 0.05) in all the homogenized treated and untreated constructs; of interest, this up-regulation was independent of the treatment modalities. *Type IV collagen* up-regulation was directly correlated with the morphological pattern of vascular tissue invasion and capillary sprouting within the macroporous spaces that characterized morphologically all treatment modalities when evaluated on day 15. Of interest, *type IV collagen* expression, although reduced, was also present on day 90, correlating with the continuous induction of bone formation sustained by capillary sprouting as seen morphologically within the treated and untreated macroporous constructs (Fig. 3). Of note, limited angiogenesis and capillary sprouting in biphosphonate zoledronate-treated samples showed a significant reduction in *type IV collagen* expression on day 60 (Fig. 3C). *Bone morphogenetic protein-2* was up-regulated on day 90 in untreated control specimens (Fig. 3A). Verapamil hydrochloride-treated samples showed down-regulation of the *BMP-2* gene on day 15, with significant up-regulation on day 90 (Fig. 3B). *Noggin* expression was significantly up-regulated on day 15 in tissue specimens of both verapamil- and zoledronate-treated samples (Fig. 3B and C). To the contrary, untreated control specimens showed down-regulation of *Noggin* at all time periods (Fig. 3A). Importantly, up-regulation of *Noggin* was more pronounced in biphosphonate zoledronate-treated specimens than in verapamil hydrochloride-treated constructs (Fig. 3C).

## Discussion

The future of the continuing evolution of biomaterials 1 is to functionalize the implanted biomaterials’ surfaces to evoke a pattern of gene expression that invocates selected tissue biology and morphological cascades 3–25. Such novel biomaterials when interacting with multipotent pleiotropic stem cells ‘niches’ induce specific molecular and tissue biology phenomena initiating regenerative responses as inductive biomaterials 2–25. Importantly, the induction of tissue formation occurs without the exogenous application of the soluble molecular signals of the transforming growth factor-β (TGF-β) supergene family 3,4.

The micro crystallinity of the implanted biomatrices, lacunae, pits and concavities cut by osteoclastogenesis together with Ca^++^ release are the driving morphogenetic and ionic cues that set into motion the induction of bone formation 28–29. Responding stem cells’ differentiation and the induction of the osteogenic phenotype are controlled by Ca^++^ release within the protected microenvironment of the secluded macroporous spaces after nano-topographical modifications of the implanted macroporous surfaces primarily by osteoclastic cells 39–42.

By harvesting samples at an earlier time period, *i.e*. 15 days, the present study has shown prominent vascular invasion and capillary sprouting within the most peripheral macroporous spaces of all the implanted macroporous constructs. This prominent and remarkable angiogenesis is molecularly initiated by the up-regulation of *type IV collagen* in treated and untreated samples. Type IV collagen is essential for the morphogenesis of the basement membrane of the sprouting and invading capillaries 17–43.

Previous results have shown that there is a temporally and spatially related sequence of tissue alkaline phosphatase expression during the developmental processes culminating in bone differentiation 3; of interest, the induction of bone differentiation has been also related to the developmental sequence of the induction of collagenous condensations, which pre-date the induction of bone formation 3,13 together with invading capillaries. These, interpreted as the ‘*osteogenetic vessels*’ of Trueta's definition 44, promote the cascade of bone differentiation.

The present iconographic digital data show how the vascular bundle supported by collagenic fibres enters and proceeds within the macroporous spaces from the most peripheral areas of the implanted construct. Classic studies have shown that the extracellular matrix components of type IV collagen and laminin bind morphogenetic proteins involved both in angiogenesis, *i.e*. basic fibroblast growth factor 45–46, TGF-β_1_ 47 and osteogenesis, *i.e*. osteogenin and OP-1 48–49. Bound angiogenic and bone morphogenetic proteins are then presented locally in an immobilized form to responding stem cells and osteoprogenitors-alike to initiate osteogenesis in angiogenesis 43–50.

The sprouting invading capillaries almost touch the calcium phosphate-based substratum as well as the osteoblasts, and release a continuous flow of paravascular/pericytic stem cells from the vascular compartment to the differentiating osteogenic compartment attached to the coral-derived biomimetic matrix 3–50. Based on the fundamental insights of Trueta 44, classic *in vitro* experiments have indicated that bone-forming cells are in contact with the basement membrane of the invading capillaries playing a key role in formation of a network of cytoplasmic processes resembling the osteocytes’ canalicular network 51. Our morphological images show the exquisite relationship between osteoblastic cells polarized on the secreted bone matrix with the invading sprouting capillary whose basement membrane components touch the polarized osteoblastic cells.

Recent papers, expanding on *in vivo* manufacturing of vascularized autogenous skeletal replacement parts 21, investigated axial vascularization to promote and enhance angiogenesis in osteoconductive matrices 52,53. A direct comparison with the reported vascular invasion within the macroporous spaces of the intramuscularly implanted coral-derived constructs is, however, difficult. Further studies in *P. ursinus* should be planned using coral-derived constructs supported by the arteriovenous loop approach 52,53 to additionally investigate the critical role of axial vascularization in the induction of bone formation by coral-derived macroporous constructs.

On day 15, biphosphonate-treated specimens showed minimal, if any, *BMP-2* expression, but up-regulation of the *Noggin* gene. More importantly, however, besides the limited bone formation as evaluated histomorphometrically, zoledronate-treated specimens showed a profoundly delayed tissue patterning with poorly constructed collagenous condensations, so critically important for the induction of bone formation 3,13. Delayed tissue patterning and poorly constructed mesenchymal tissue condensations, and later cell differentiation and osteogenesis, are as a result of the lack of osteoclastic-driven nano-patterned geometric configurations, which result in Ca^++^ release with the expression of the osteogenic phenotype 7–57. Importantly, lack of osteoclastic activity does result in limited, if any, Ca^++^ release; a reduction in Ca^++^ release results in lack of angiogenesis and stem-cell differentiation 31–58, thus effectively controlling *ab initio* the induction of bone formation.

The importance of tissue patterning and the induction of mesenchymal tissue condensations are critically shown by the lack of osteoclastic activity in zoledronate-treated specimens. In previous experiments using coral-derived macroporous constructs, morphological and immunocytochemistry analyses have suggested the critical morphogenetic role of the morphogenesis of mesenchymal tissue condensations against the implanted substratum, further indicating that condensations pre-date the induction of bone formation 3,13.

Cellular and extracellular matrix condensations are pivotal in the development of skeletal tissues 59. Zoledronate-treated specimens showed a profound delay in tissue patterning and morphogenesis; molecularly, this tissue patterning disarray is pre-dated by *Noggin* up-regulation together with minimal, if any, *BMP-2* expression on day 15. The data indicate the critical role of BMPs in setting tissue patterning and morphogenesis before the induction of bone formation.

Zoledronate-treated specimens showed *Noggin* up-regulation on day 15 as compared with untreated control samples. Noggin prevents BMP binding to the BMP receptor 60 and as such inhibits bone formation. The loss of osteoclast binding resulted in a temporary up-regulation of *Noggin* on day 15; this up-regulation generated minimal bone formation by induction on days 60 and 90. Our results further suggest that the biphosphonate zoledronate’ effect is temporary, possibly degraded by days 60 and 90, with down-regulation of *Noggin,* resulting in delayed and minimal bone induction yet again at the periphery of the implanted macroporous constructs only.

When the L-type voltage-gated ion channel for Ca^++^ was blocked by pre-loading the macroporous constructs with verapamil hydrochloride, morphological and histomorphometric analyses showed limited bone formation. Tissue patterning was not as disorganized and delayed as in zoledronate-treated specimens. Verapamil-treated samples showed down-regulation of *BMP-2* together with up-regulation of *Noggin* on day 15, suggesting that Ca^++^ regulates gene expression 61 and thus controlling the induction of bone formation. Limited induction of bone formation on days 60 and 90, although preferentially at the periphery of the implanted macroporous constructs, may indicate degradation of the implanted verapamil-hydrochloride. Lack of active verapamil with delayed bone induction on day 90 is accompanied by recovery of *BMP-2* expression and notably by *Noggin* down-regulation on days 60 and 90.

The acidic microenvironment of the implanted intramuscular pouch may also result in dissolution of hydroxyl apatite and carbonate bound calcium resulting in higher extracellular calcium concentrations within the protected macroporous spaces of the coral-derived constructs. Of note, implanted macroporous calcium carbonate constructs were partially converted to 7% hydroxyapatite, which could possibly release Ca^++^ at minor intramuscular pH changes during healing 28,29. The biological effects of several ions have been well documented, including the induction of angiogenesis by triggering the secretion of angiogenic morphogens 62. Underlying cellular migration and differentiation are exceedingly complex intracellular events focused on cytosolic flooding by calcium ions either from the endoplasmic reticulum or the extracellular domain *via* calcium channels in the cell membrane 63.

The most likely triggering events for the spontaneous induction of bone formation are, on the one hand, ionic with Ca^++^ release in the protected microenvironment of the macroporous spaces and, on the other, cellular and morphological whereby osteoclastic-based nano-patterned surface modifications with Ca^++^ release differentiate locally responding stem cells into secreting osteoblasts. The coral-derived macroporous constructs are thus richly vascularized because of the calcium gradient; vascularization with capillary sprouting populate the macroporous spaces with mesenchymal stem cells of vascular/paravascular derivation capable of responding both to the newly carved functionalized nano-patterned topographies 7–41 and to the calcium ions concentration peak 32–36. *In vitro*, the proliferation of human vascular smooth muscle cells is inhibited by the addition of the L-type voltage-gated calcium channel blocker verapamil 42. Verapamil blocks extracellular calcium ingress to cytosol of several cells involved in the angiogenic response and irreparably halts the nascent angiogenic phase 42.

The formation of bone by osteoblasts and its remodelling by osteoclasts are a closely integrated homeostatic system 68. The bone remodelling cycle is the cumulative result of the intimate and complex interaction of the cellular components of the basic multicellular unit (BMU) 23,24. Mineralized surfaces including hydroxyapatite and tricalcium phosphate substrata recruit and induce osteoclast formation 70–71. The mesenchymal cellular condensations cued by the nanotopography of the implanted macroporous surfaces, together with the Ca^++^ concentration peak facilitated by the protected microenvironment of the secluded macroporous spaces, will attract circulating osteoclast precursor cells (OCP) to populate the coral-derived macroporous surfaces and initiate the osteogenic activity of the BMU by setting the activation phase of resorption and by recruiting osteoblastic precursor cells. The nanotopographical geometric configurations set by osteoclastogenesis together with Ca^++^ ions release differentiate myoblastic/paravascular and/or pericytic stem cells into osteoblastic cell lines.

The inductive surface microstructure of various hydroxyapatite-based macroporous constructs is the common denominator of materials endowed with the striking prerogative of initiating *de novo* bone formation by induction 72. Surface microstructure resulting from micro surface porosity may be drastically affected by sintering, thereby affecting the induction of bone formation when implanted in heterotopic sites 72.

Differentiated osteoblasts will express and secrete osteogenic proteins of the TGF-β supergene family to be embedded into the matrix initiating the induction of bone formation as a secondary response. There are temporally and spatially related phenotypic transitions ultimately culminating in the differentiation of osteoblastic-like cells and the induction of bone formation 3. The angiogenic response penetrating the macroporous spaces of the coral-derived constructs is a prominent morphological feature. This was shown by the pattern of alkaline phosphatase activity of the invading capillaries 3 as well as by the expression of *type IV collagen* on day 15 in the present study. It is likely that the temporal induction of *type IV collagen* and angiogenesis is initiated long before the inhibitory activities of both the calcium ion blocker, verapamil hydrochloride and the osteoclast inhibitor, biphosphonate zoledronate.

Finally, the presented study is the only experiment *in vivo* correlating tissue morphology with the gene expression of the generated tissues by macroporous constructs at different time-points and in a non-human primate model 73. The nascent tissue induction and tissue patterning as seen morphologically have been studied by qRT-PCR; because of the lack of *in vivo* studies, we still do not know the level and the extent of tissue induction changes, which impact gene expression. The opposite is perhaps more true and important: we have not grasped as yet the extent of gene expression changes necessary to set into motion the quantity and quality of regenerated tissue morphology particularly in primate species. The knowledge of this fine molecular/morphological tuning would be exceedingly complicated to gather from *in vivo* studies, particularly in primates, but may well be the next boundary in regenerative medicine and tissue engineering.

Lastly, the harvested tissue for the molecular biology studies may not fully represent or capture the quantity and quality of tissue morphology changes that occur at the microenvironment level, calling for a more sophisticated approach in studying the events at the cell/matrix interface.

In conclusion, this non-human primate study establishes the critical role of osteoclastogenesis to regulate the induction of bone formation by coral-derived biomimetic matrices. It further shows the regulatory role of Ca^++^ and L-type voltage-gated ion channels in controlling the induction of bone formation. Osteoclastogenesis is required to set nano-patterned geometric topographies, which, together with Ca^++^ release, induce angiogenesis and capillary sprouting, cellular differentiation, osteoblastic cell attachment and synthesis to the implanted biomimetic matrix. Expression of *BMPs* is followed by the embedding of the secreted gene products into the implanted macroporous surfaces inducing bone formation as a secondary response. The induction of *BMPs,* together with *Noggin* controlling the extent of bone differentiation by induction, indicates that the induction of bone formation by coral-derived biomimetic matrices is initiated and maintained *via* the *BMPs* pathway.

The induction of bone formation as initiated by macroporous coral-derived constructs might require fewer amounts in hBMPs/hOPs when applied in clinical contexts.
